# In Memoriam: Washington C. (Wash) Winn Jr. (1941–2011)

**DOI:** 10.3201/eid1712.IM1712

**Published:** 2011-12

**Authors:** David H. Walker, Rocco LaSala, Bobbi Pritt, Elmer Koneman, J. Michael Miller

**Affiliations:** Associate Editor, Emerging Infectious Diseases (D.H. Walker);; University of Texas Medical Branch, Galveston, Texas, USA (D.H. Walker);; West Virginia University, Morgantown, West Virginia, USA (R. LaSala);; Mayo Clinic, Rochester, Minnesota, USA (B. Pritt);; University of Colorado, Denver, Colorado, USA (E. Koneman);; Centers for Disease Control and Prevention, Atlanta, Georgia, USA (J.M. Miller)

**Keywords:** Washington C. Winn, viruses, bacteria, microbiology, in memoriam, obituary

Washington (Wash) C. Winn Jr., (Figure) died suddenly and unexpectedly on July 3, 2011. A remarkable Renaissance man; a warm, humane person; and outstanding academic physician and scientist, Dr. Winn is remembered vividly as a contributor to understanding of emerging infectious diseases; a contemporary, efficient diagnostic clinical microbiologist; and a treasured educator. Born in Richmond, Virginia, on April 2, 1941, he was a true scholar, graduating magna cum laude with honors in English from Yale University in 1963. All who have been a co-author with, been edited by, or shared an educational venue with him have experienced a captivating creativity, mastery of expression, and improvement in one’s own communication through his skills with the language.

**Figure:**
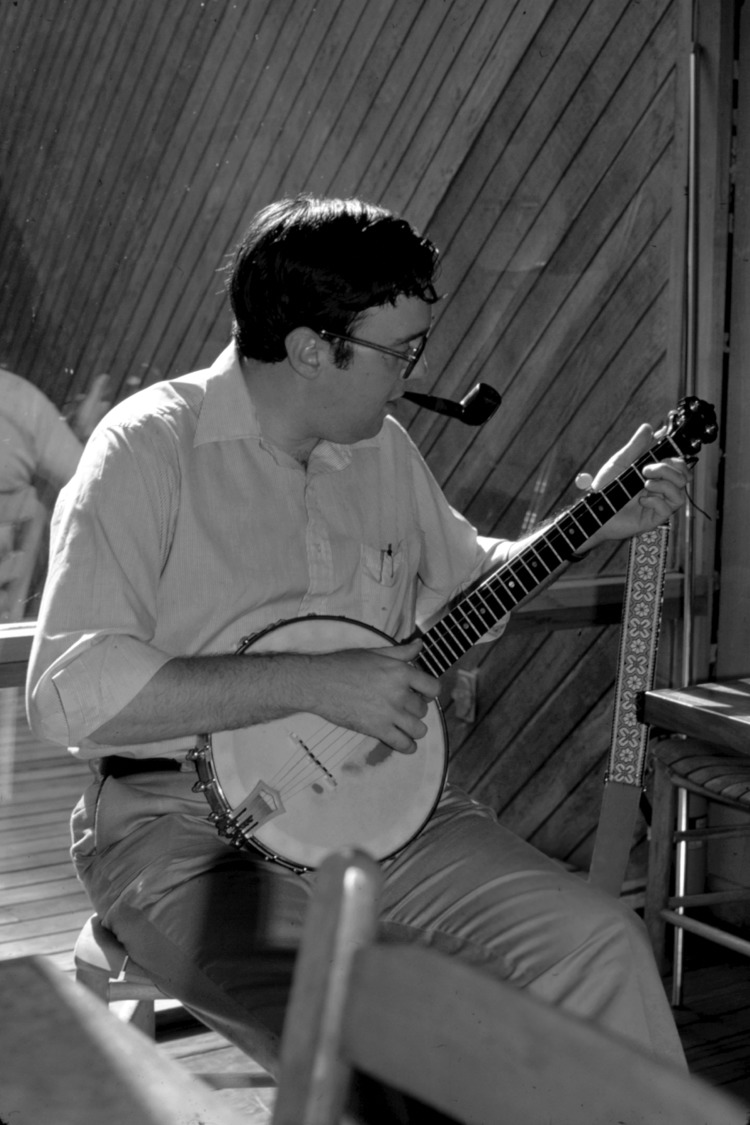
Washington C. Winn, Jr.

Dr. Winn earned his doctor of medicine degree from the University of Virginia (1967) and deepened his experience as a physician during an internship in medicine at Tufts–New England Medical Center where he received the Medical Intern Award. Subsequently, he served as a resident in pathology at Washington University in St. Louis (1968–1970). Dr. Winn also earned a master’s degree in business administration at from the University of Vermont in 1993.

A critical period in his life (1970–1973) occurred when he was a medical officer at the Center for Disease Control in Atlanta. He served in the Arbovirus Reference Unit and the Viral Pathology Branch, Division of Viral and Rickettsial Diseases, where he attributed the mentoring influence of Frederick A. Murphy on the subsequent direction of his professional career. His research on rhabdoviruses and arenaviruses led to his first 7 scholarly publications, including contributions to our knowledge of the pathology of Lassa fever, an archetypal emerging infectious disease. His colleagues of that era remember him as “a warm and wonderful guy and a true Virginia gentleman” (Thomas Monath, pers. comm., 2011), “who lived a life of real grace in everything he did or thought” (Karl Johnson, pers. comm., 2011).

After 4 years as assistant professor of pathology at the University of Virginia (1973–1977), Dr. Winn moved to the University of Vermont where he served as director of the clinical microbiology laboratory for 34 years. An outbreak of Legionnaires’ disease in the hospital led to investigations of this disease, which was emerging at the time. As principal investigator of a National Institutes of Health grant, he contributed to our knowledge of the pathology of *Legionella pneumoniae* and the basic pathogenesis of legionellosis and to methods for the clinical microbiologic diagnosis of the disease, with 29 peer-reviewed publications on these topics.

Dr. Winn then made a conscious positively motivated decision to focus his career on clinical microbiology, with resulting tremendous benefits to the field nationally and internationally and to patients in his institution. His clinical microbiology laboratory at Fletcher Allen Health Care, the clinical program of the University of Vermont, was a model of success. Through the decades, he assembled an exceptional technical staff. He had honed laboratory policies and optimized procedural details to such a degree that problems were essentially nonexistent. He kept the laboratory up to date with the latest technologies. In 2006, his goal was to incorporate real-time PCR into the laboratory—something that was not available in any clinical or anatomic pathology laboratory at the institution. Within just 5 years, he transitioned an essentially nonmolecular test menu to one with a full spectrum of qualitative and quantitative real-time PCRs that encompassed locally developed and Food and Drug Administration–approved assays.

His national contributions to clinical microbiology included membership on the Microbiology Test Committee of the American Board of Pathology and service as chairman, vice chairman, and member and advisor of the Microbiology Resource Committee of the College of American Pathologists. In these roles, he is remembered not only as a knowledgeable scientist and diagnostician but also as a person of character and genuine concern for those with whom he worked. He was always willing to share what he knew in an unselfish way that was endearing to everyone who learned from him.

Another great contribution to the field of clinical microbiology was Dr. Winn’s involvement in the influential textbook *Color Atlas and Textbook of Diagnostic Microbiology*, in which he joined Elmer Koneman as co-author of the third edition (1988) (*1*). He wrote chapters on virology, antimicrobial drug susceptibility testing, and new technologies in the diagnosis of infectious diseases in the next 3 editions and added a section on ectoparasites to the parasitology chapter in the fifth edition. He became chief editor of the sixth edition (2006) (*2*), which in the opinion of Dr. Koneman was the crown jewel in the life of the book (E. Koneman, pers. comm.).

Memories of Dr. Winn are indelible and include his extraordinary wealth of knowledge and passion for opera, particularly when the scenes and music were linked to medical practice, such as the final arias sung by Violetta (*La Traviata*) and Mimi (*La Boheme*) before dying of tuberculosis. He will be remembered for his joy in playing the banjo and fiddle, love of good wines and diverse cuisines, learning the German language and Egyptian hieroglyphics, expressing staunch Republican opinions in a sea of liberal colleagues, and cultivating bonsai and ancient varieties of roses. His wisdom, humor, insight, honesty, and kindness will be missed tremendously. Dr. Winn was also a member of the Emerging Infectious Diseases review panel.
